# The Impact of Diabetes Mellitus in COVID-19: A Mechanistic Review of Molecular Interactions

**DOI:** 10.1155/2020/5436832

**Published:** 2020-11-17

**Authors:** Habib Yaribeygi, Thozhukat Sathyapalan, Tannaz Jamialahmadi, Amirhossein Sahebkar

**Affiliations:** ^1^Research Center of Physiology, Semnan University of Medical Sciences, Semnan, Iran; ^2^Academic Diabetes, Endocrinology and Metabolism, Hull York Medical School, University of Hull, UK; ^3^Department of Food Science and Technology, Quchan Branch, Islamic Azad University, Quchan, Iran; ^4^Department of Nutrition, Faculty of Medicine, Mashhad University of Medical Sciences, Mashhad, Iran; ^5^Biotechnology Research Center, Pharmaceutical Technology Institute, Mashhad University of Medical Sciences, Mashhad 9177948564, Iran; ^6^Neurogenic Inflammation Research Center, Mashhad University of Medical Sciences, Mashhad, Iran; ^7^School of Pharmacy, Mashhad University of Medical Sciences, Mashhad, Iran

## Abstract

The ongoing pandemic of COVID-19 is now the major issue in global health. Evidence implies that patients with diabetes are at a higher risk of severe disease or death due to COVID-19 than individuals without diabetes. However, the underlying mechanism for this differential effect in individuals with and without diabetes is not clearly understood. We have reviewed the pathophysiological pathways which may facilitate the entry of virus or an increase in its infectivity in host cells in the diabetic milieu. We suggest that the preexisting pathological pathways in patients with poorly controlled diabetes increase the risk of infectivity and are responsible for the higher levels of tissue injury and death in patients with diabetes.

## 1. Introduction

Coronavirus disease 2019 (COVID-19) is an infectious disease developed by severe acute respiratory syndrome coronavirus-2 (SARS-CoV-2) [1]. This disease was initially detected and reported in December 2019 in Wuhan city (China) and then spread rapidly to other cities and countries, resulting in the ongoing pandemic in more than 200 countries worldwide [1–3]. It is now the single most crucial issue in global health, and many researchers are looking for effective therapeutic and preventive agents for the treatment of COVID-19 worldwide [3]. Although no vaccine or therapeutic agent has yet been found to be effective for the management of COVID-19 yet, however, there is an increasing body of evidence suggesting it has strong associations with certain clinical conditions such as diabetes mellitus (DM) [2, 3]. While the overall rate of death is estimated to be lower than 6 percent among patients with COVID-19 disease [4], but patients with DM are at a higher risk [2]. Ongoing studies suggest that patients with diabetes who have poorly controlled glycemia have around four times higher death rate and longer length of hospitalization compared to patients without DM [5, 6]. Although more evidence is needed to confirm these findings, it has been shown that DM increases the risk of COVID-19 complications [6]. However, the specific molecular interactions between DM and COVID-19 are not understood well. In the current study, we will discuss possible molecular interactions between DM and COVID-19 for potentially developing some novel preventive and therapeutic agents against COVID-19 in patients with DM.

### 1.1. COVID-19

Coronaviruses are a family of viruses with a genome size of around 26 to 32 kilobases and a size of 80–220 nm in diameter, making them the largest among RNA viruses [7, 8]. They are surrounded and enveloped by a fatty outer layer and commonly have spherical shapes with a crown or “corona” of club-shaped spikes on their surface [9]. These spikes are responsible for the virus-receptor binding in cell surface [9]. Arthur et al. in the 1930s were the first to report a case of acute respiratory infection in chickens and suggested that it may be related to a specific type of viruses [10]. Later, Fred and coworkers in 1937 isolated and cultivated the coronavirus for the first time in the lab [11]. In the 1960s, the first human coronavirus was discovered from a boy who had a novel type of cold [9, 12, 13]. Coronavirus gains entry by binding to the cell surface receptors via the S protein (spike), which is then cleaved into two functional subunits known as S1 and S2 [14]. Upon binding of S1 to the specific receptor, a conformational change is triggered in the S2 subunit, followed by viral delivery into the cytoplasm after several sequential molecular steps [14]. Until today, seven types of viruses from this family are found to infect humans which are SARS-CoV, MERS-CoV (Middle East respiratory syndrome coronavirus), SARS-CoV-2, HKU1 (Human coronavirus 1), NL63 (Human coronavirus NL63), OC43 (Human coronavirus OC43), and 229E (Human coronavirus 229E) [15]. While the first three viruses are linked to severe respiratory infections, others commonly cause mild upper respiratory symptoms [15].

SARS-CoV-2 is a single-stranded and an important member of the coronavirus family which was recently discovered in late 2019 as responsible for the COVID-19 pandemic [16]. It was discovered first in Wuhan, the capital of China's Hubei province [16, 17]. COVID-19 is now rapidly spreading in more than 200 countries, and as of May 10, 2020, there are 4.02 million confirmed cases of COVID-19 and 279,000 deaths reported worldwide [18]. Fever, fatigue, cough, shortness of breathing, chest pain, and loss of smell are the common symptoms of COVID-19 [19]. However, in severe cases, it can cause severe lower respiratory tract symptoms and low oxygen saturation in the blood resembling acute respiratory distress syndrome and will need mechanical ventilation [5, 20]. The World Health Organization (WHO) confirmed that the COVID-19 outbreak is a Public Health Emergency of International Concern on January 30, 2020, and a pandemic on 11 March 2020 [21]. At present, we have no specific vaccine or antiviral treatment available to manage COVID-19. The primary strategy for managing COVID-19 currently is only symptomatic treatments and supportive care in addition to isolation, and experimental therapies [5].

The lungs are the primary organ for SARS-CoV-2 [1]. However, it may also infect the gastrointestinal tract, central nervous systems, and cardiovascular system [1, 22, 23]. SARS-CoV-2 binds to the host cells via linking to angiotensin-converting enzyme type 2 (ACE2), which is responsible for catalyzing the hydrolysis of angiotensin II (Ang II) into angiotensin (1–7) (a vasodilator) [24]. This enzyme is abundantly expressed on the alveolar type II cells of the pulmonary tissues but also exists in other types of the cells such as neurons and myocardial cells [1, 22, 23]. Hence, blocking the ACE2 expression and activity may provide protective effects against COVID-19 infection [25]. However, there is evidence suggesting that ACE2 inhibitors may increase the risk of COVID-19 complications [26] and needs more research.

### 1.2. COVID-19 and Diabetes

As stated earlier, DM increases the complications of COVID-19 and the risk of COVID-19 related mortality [5, 6]. Current evidence demonstrates that patients with DM are more likely to experience severe symptoms and complications than patients without DM due to COVID-19 [5, 6]. One hypothesis is that hyperglycemia facilitates the virus entry into the cells since ACE2 and virus both need glucose for their function [27]. Although to understand the exact interactions between COVID-19 and DM requires more research, we have reviewed the potential molecular mechanisms involved from a cell biology point of view.

### 1.3. Inflammation and Immune System Activation

Inflammation is a biological reaction of the innate immune system against various harmful stimuli. It is primarily considered as a protective mechanism for the removal of injurious stimuli such as toxins, irritants, damaged cells, and pathogens for maintaining homeostasis [28, 29]. Inflammatory responses are complex molecular events involving various types of immune cells (lymphocytes, monocytes, and mast cells), and an extensive series of inflammatory mediators such as TNF-*α* (tumour necrosis factor-alpha), IL (interleukin), MMPs (matrix metalloproteinase), MCP-1 (monocyte chemoattractant protein-1), nf-*κ*b (nuclear factor kappa b), TGF-*β* (transforming growth factor-beta), adhesion molecules, TLRs (toll-like receptors), adipocytokines, CRP (C-reactive protein), and INF-*γ* (interferon-gamma) [28, 30–35]. Acute inflammation is the initial phase of immune system activation for eliminating various harmful factors from the tissues [28]. But sometimes, it may take longer than the physiologic state and become chronic inflammation, especially in states of dysregulation of immune responses in conditions such as DM [29]. DM is associated with chronic low-grade inflammation in the body, and patients with diabetes have higher circulatory levels of cytokines [32, 36–39]. Also, these cytokines are intimately involved in the pathophysiology of various diabetic complications and increase the risk of diabetes-induced tissue damages [39, 40].

COVID-19 is a viral infection characterized by storms of inflammatory responses and higher levels of circulatory cytokines [41, 42]. These storms are frequent in patients with severe stages of COVID-19 [42]. Clinical evidence demonstrates that COVID-19 patients have impaired immune system activity, especially with critical illness [42, 43]. Many of the COVID-19 victims appear to be harmed more by their immune system hyperactivity. Therefore, lowering the inflammatory response is a potential strategy for the management of COVID-19 [43]. Due to the presence of chronic inflammation in DM, it could be a potentiator of inflammatory responses and increase the likelihood of inflammation storms in COVID-19 patients [44]. Patients with diabetes associated with COVID-19 potentially have a higher rate of inflammatory responses [44]. Guo et al. recently evaluated 174 patients with confirmed COVID-19 and found that in patients with diabetes the levels of various inflammatory cytokines including IL-6, CRP, serum ferritin, coagulation index, and D-dimer were significantly higher than nondiabetic COVID-19 patients. This finding suggests that patients with DM are more susceptible to inflammatory storms leading to more severe degrees of the respiratory infections [44]. Also, previous studies on coronaviruses demonstrated that inflammatory milieu increases the likelihood of infection with coronaviruses [45–47]. It has been shown that host cell condition has significant effects on the infectivity and pathogenesis of various pathogens such as SARS-CoV-2 [48–50]. Thus, having low-grade chronic inflammation is commonly seen in patients with diabetes increase the likelihood of inflammatory storms resulting in more severe tissue damage [45–47]. Therefore, inflammation could be a possible link between DM and COVID-19 progression [45–47].

#### 1.3.1. Oxidative Stress

Oxidative stress refers to the imbalance between free radical species and the potency of antioxidant defence systems in favor of the free radicals. It plays an essential role in the pathophysiology of various complications of DM as well as viral respiratory disorders [51–54]. In addition to damaging various biological molecules in the respiratory tract, this pathologic milieu initiates and progresses other molecular mechanisms involved in respiratory infections such as uncontrolled apoptosis or necrotic processes [53, 55]. Using antioxidant supplements could sometimes help as adjuvant therapy for the management of certain respiratory infections [55]. It has been shown that DM is associated with the generation of excess free radicals [30, 56]. Uncontrolled DM induces oxidative stress via at least ten molecular mechanisms such as mitochondrial dysfunction, weakening the cellular antioxidant elements, glucose autoxidation, glycation and related pathways, lipids peroxidation, activation of free radical generator enzymes, polyol (sorbitol) pathway, protein kinase C isoforms, hexosamine pathway, and redox state changes [30]. This oxidative milieu is implicated in most viral infections and may increase the pathogenicity of viruses such as coronaviruses [48, 57, 58]. Since SARS-CoV-2 exploits the host cell machinery for their replication and spread, the environment of the host cells is a crucial determinant for the infectivity of invader pathogens, and oxidative milieu is one of the main factors facilitating coronavirus replication in the host cells [48–50]. Also, oxidative stress activates other pathophysiologic pathways such as inflammation and necrosis and promotes downstream molecular mechanisms such as mitogen-activated protein kinase (MAPK) which intensify the progression of viral infection in the tissue [50]. Moreover, oxidative stress may facilitate coronavirus entry into the cells via modulating their route of entry [50]. For example, free radicals have significant impacts on the transmembrane protease serine 2, a primary protein involved in nonendosomal virus entry, and alter its distribution [59, 60]. Hence, oxidative stress in host cells is a key determinant factor for the coronavirus entry, replication, and pathogenesis [61].

Wu et al. in 2008 reported that oxidative stress increases the risk of coronavirus infection [48]. They demonstrated that G6PD (glucose-6-phosphate dehydrogenase) deficient cultured cells are more susceptible to coronavirus-induced infection compared to normal cells [48]. G6PD is a crucial metabolic enzyme involved in maintaining normal cellular redox state [48, 62]. In addition, Imai et al. in 2008 found that severe respiratory infections due to coronaviruses are closely related to the activation of oxidative stress machinery in the cells [46]. They have shown that mutant mice resistant to oxidative stress (TLR4 (Toll-like receptor 4) knockout) were at a lower risk of coronavirus-induced severe respiratory infection than an intact animal. This suggests that modulating oxidative stress could provide potential preventive or therapeutic effects in patients infected with coronavirus [46]. Although more research is needed, current evidence highly suggests that modulating the oxidative stress in patients with diabetes could be potentially useful in reducing the risk of viral respiratory infections such as COVID-19 [63–67].

#### 1.3.2. Glucotoxicity

The toxic impact of excess amount of glucose on various metabolic pathways, which is commonly seen with uncontrolled DM, is known as glucotoxicity [68, 69]. This pathologic process deranges the glucose homeostasis toward pathways such as polyol, hexosamine, or sorbitol pathways in hyperglycemic milieu [68]. Also, it is commonly accompanied with upregulation of proteins involved in cellular injuries such as proapoptotic and death receptors, caspases and TLRs, and activation of molecular pathways such as c-Jun NH2-terminal kinase-1 (JNK), Bax/Bcl2, and PKR-like ER kinase (PERK) in various tissues [68, 69]. Glucotoxicity is also able to induce other pathophysiologic pathways as oxidative stress, inflammation, fibrosis, apoptosis, and necrosis in multiple tissues [69]. Hence, it is now accepted that glucotoxicity has major indirect roles in hyperglycemia-dependent histological damages in various tissues including the lungs and respiratory tract [70–72].

Although there is not enough direct evidence yet, we suggest that COVID-19 induces tissue injuries in the diabetic milieu is at least partly associated with glucotoxicity which can onset and promote other pathophysiologic mechanisms [73]. In a recent study, Codo and coworkers reported that elevated levels of glucose favor COVID-19 infections via a hypoxia-inducible factor-1*α*- (HIF-1*α*-) dependent mechanism [74]. They suggested that patients with diabetes are more prone to SARS-CoV2 due to toxic effects of hyperglycemia [74]. Also, we suggest that higher severity of tissue injuries in the respiratory system which occurs in patients with DM infected with SARS-CoV2 are closely related to the toxic effects of hyperglycemia that induces various injurious pathways that damage the infected host cells [48–50, 73]. Therefore, maintaining the homeostatic state of the glucose in these patients could markedly prevent the severity of COVID-19 infection and reduce the rate of injury and death by preventing glucotoxicity-induced cellular damages.

#### 1.3.3. ER Stress

Endoplasmic reticulum (ER) is a cellular organelle which is involved in the synthesis and export of biomolecules such as lipids, proteins, and carbohydrates. It is also involved in the processing and maturation of these biomolecules by folding, glycosylation, and disulfide bond formation [75]. This vital organelle shuttles biomolecules to their correct destination for maintaining the cellular homeostasis [48]. ER-resident chaperons, including GRP78 (glucose-regulated protein 78) and GRP94, play a remarkable role in these activities [76]. Any impairment in ER activity is related to the aggregation of un/misfolded proteins in the ER lumen, resulting in a pathologic state of “ER stress” [77]. Once ER stress develops, unfolded protein response (UPR) is activated to maintain homeostasis in the cells through downregulation of global proteins, upregulation of proteins involved in folding, increasing chaperone levels, and promoting the degradation of misfolded or unfolded proteins via proteasome and autophagy [75]. In a prolonged ER, stress exceeding UPRs capacity, cells activate several intracellular signalling pathways that may lead to cell suicide through the apoptosis [78].

ER stress is a common event in the diabetic milieu [79, 80]. It is closely linked to various complications associated with diabetes [80]. On the other hand, it has potent interactions with coronavirus activity [81, 82]. Versteeg et al. in 2007 demonstrated that coronavirus induces ER stress via upregulation of spike proteins and promotes the cellular entry of the virus [81]. Fung et al. in 2014 suggested that ER stress promotes viral replication and increases the rate of coronavirus infection [14]. The ER stress increases the infectivity of coronaviruses and raises the pathogenicity of these viruses [14]. The cells overexpressing S2 subunit of SARS-CoV spike also upregulate the GRP94 and GRP78 chaperones [83, 84]. Also, other biomarkers of ER stress such as HERPUD1 (homocysteine-inducible, ER stress-inducible, ubiquitin-like domain member 1) were upregulated in murine cells infected with SARS-CoV [81], indicating that ER stress has a crucial role in the pathogenicity of the coronaviruses [14]. Evidence highly suggests that ER stress is associated with higher infectivity of coronaviruses and increases their pathogenic potency [81, 85]. SARS-Co viruses can modulate different molecular pathways involved in the UPR, such as PKR-like ER protein kinase, PERK, eIf2-*α* (Eukaryotic Initiation Factor 2) phosphorylation, IRE1 (inositol-requiring protein-1), and ATF6 (activating transcriptional factor-6) [75, 85]. Although there is no direct evidence yet, one can speculate that higher pathogenicity of SARS-CoV2 in patients with DM is at least partly linked to preexisting ER stress in these patients which might promote virus entry and pathogenicity [82, 86, 87]. This suggests that UPR could be a potential therapeutic target for developing novel treatments against SARS-CoV2.

#### 1.3.4. RAAS System

Renin-angiotensin-aldosterone system (RAAS) is a hormonal system responsible for water and electrolytes homeostasis as well as maintaining systemic vascular resistance [88]. This system is triggered by releasing renin from the kidneys followed by cleaving angiotensinogen into angiotensin I (Ang I) by renin [88]. Ang I is subsequently converted to angiotensin II (Ang II) by the angiotensin-converting enzyme (ACE) predominantly on the surface of vascular endothelial cells of the lungs (as well as proximal renal tubules) [88, 89]. While ACE catalyzes this conversion, ACE2 counter this activity by increasing Ang 1–7 and decreasing the active levels of Ang II [89]. Ang II is the main final effectors of this system and a potent vasoconstrictive peptide [88, 89]. It acts mainly via binding with two kinds of receptors called type 1 (AT1) and types 2 (AT2) [88]. Also, it induces the release of aldosterone, another hormone involved in the electrolyte homeostasis [88].

SARS-CoV2, which is responsible for the recent pandemic of COVID-19, has close interactions with RAAS activity [90]. SARS-CoV2 enters the cells via binding with spike (S) protein of ACE2 receptors, and hence, pharmacologic agents modulating RAAS activities have been proposed for the management of COVID-19 pandemic in recent studies [90]. It has been shown that SARS-CoV2-infected patients are associated with different degrees of alteration in RAAS activities [26, 91]. On the other hand, patients with DM have some degree of changes in RAAS functions and widely use ACE inhibitors or ARBs (Angiotensin receptor blockers) to prevent or treat diabetes-induced vascular disorders [92, 93]. Also, these drugs repeatedly showed beneficial effects against pulmonary complications and the risk of pulmonary infection in hypertensive patients with altered RAAS function compared to normotensive individuals [94–97]. Thus, it could be hypothesized that patients with DM are more likely prone to more severe degrees of COVID-19 at least partly due to their altered RAAS functions which may facilitate virus entry into the cells. However, there are some conflicting results suggesting ACE inhibitors or ARBs have no association with the risk of COVID-19 infection [26, 98–101]. Therefore, pharmacologic agents targeting RAAS could potentially reduce the severity of SARS-CoV2 in patients with DM, but more clinical studies are required.

#### 1.3.5. Apoptosis

Programmed cell death or apoptosis is a cellular event which occurs physiologically in various processes such as growth, maturation, and migration, but in uncontrolled and pathologic states, exerts histological damages. [102]. Along with necrosis and fibrotic processes, apoptosis is responsible for most types of histological injuries and tissue dysfunctions due to cellular death [103]. Apoptosis is a complicated cellular process and is under the influences of various stimuli [102]. Experimental and clinical data have been well confirmed that DM is a potent upstream event for apoptosis, and diabetic milieu is commonly associated with higher levels of apoptosis dependent cellular death [104–106]. Patients with poorly controlled hyperglycemia have higher proapoptotic factors as well as apoptosis dependent cell death in kidneys, liver, lungs, and brain [107–109].

The apoptosis also plays essential roles in coronavirus dependent tissue injuries [14, 110]. It is intimately involved in cellular death and tissue damages that occur during coronavirus respiratory tract infection [14]. Hence, it may be other possible explanation for the finding that patients with DM infected with SARS-CoV2 experience more severe tissue injuries and cellular death compared to patients without DM. We suggest that preexisting higher levels of apoptosis in poorly controlled patients with DM with higher expression of proapoptotic factors promote apoptosis-induced cell death. However, at present, this is a hypothesis and needs further research.

#### 1.3.6. Other Possible Mechanisms

In addition to the abovementioned underlying mechanisms, other possible pathways with limited evidence such as mitochondrial dysfunction in patients with DM may be involved [14, 111–114]. A mitochondrial malfunction is a common event in poorly controlled DM [115]. Mitochondrial malfunction also occurs in viral infections such as with coronaviruses [113]. This could be a possible link between DM and severity of COVID-19. Additionally, increase risk of fibrosis present in both DM and COVID-19 patients is another potential link [116]. However, we have only limited available data currently.

## 2. Conclusion

**Figure 1:**
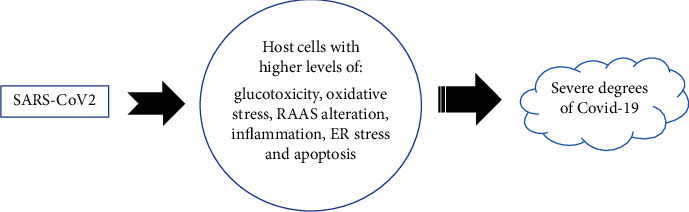
Schematic picture of our hypothesis on the mechanisms by which diabetes intensifies COVID-19 severity.

It has now been recognized that host cell condition has a significant role in the infectivity and determines pathogenicity of viruses such as SARS-CoV2. We have reviewed the potential molecular mechanisms on why patients with DM are at a higher risk of severe COVID-19 than infected individuals without DM (Figure 1). We suggest that preexisting pathophysiological pathways in patients with poorly controlled DM directly or indirectly increases the pathogenicity of SARS-CoV2. Although we currently have only limited experimental and clinical evidence, further studies on these pathways could potentially help finding more effective pharmacological agents against COVID-19 for patients with diabetes.
